# Bradykinin: Inflammatory Product of the Coagulation System

**DOI:** 10.1007/s12016-016-8540-0

**Published:** 2016-04-28

**Authors:** Zonne Hofman, Steven de Maat, C. Erik Hack, Coen Maas

**Affiliations:** 1 Laboratory of Clinical Chemistry and Hematology, University Medical Center Utrecht, Utrecht, The Netherlands; 2 Laboratory of Translational Immunology, University Medical Center Utrecht, Utrecht, The Netherlands

**Keywords:** Angioedema, HAE, Histamine, Bradykinin, Factor XII, Plasmin, D-dimer

## Abstract

Episodic and recurrent local cutaneous or mucosal swelling are key features of angioedema. The vasoactive agents *histamine* and *bradykinin* are highly implicated as mediators of these swelling attacks. It is challenging to assess the contribution of *bradykinin* to the clinical expression of angioedema, as accurate biomarkers for the generation of this vasoactive peptide are still lacking. In this review, we will describe the mechanisms that are responsible for *bradykinin* production in hereditary angioedema (HAE) and the central role that the coagulation factor XII (FXII) plays in it. Evidently, several plasma parameters of coagulation change during attacks of HAE and may prove valuable biomarkers for disease activity. We propose that these changes are secondary to vascular leakage, rather than a direct consequence of FXII activation. Furthermore, biomarkers for fibrinolytic system activation (i.e. *plasminogen* activation) also change during attacks of HAE. These changes may reflect triggering of the *bradykinin-*forming mechanisms by *plasmin*. Finally, multiple lines of evidence suggest that neutrophil activation and mast-cell activation are functionally linked to *bradykinin* production. We put forward the paradigm that FXII functions as a ‘sensor molecule’ to detect conditions that require *bradykinin* release via crosstalk with cell-derived enzymes. Understanding the mechanisms that drive *bradykinin* generation may help to identify angioedema patients that have *bradykinin*-mediated disease and could benefit from a targeted treatment.

## Introduction

Angioedema is characterized by local, non-itchy, cutaneous or mucosal edema that lasts for hours up to a few days. Angioedema is by definition a pathophysiologic process involving increased permeability of the vascular endothelial lining of small blood vessels (mostly post-capillary venules). The clinical picture of angioedema is well-defined, but the underlying mechanisms responsible for the swellings are not fully understood. Two vasoactive agents are implicated in mediating swelling attacks in angioedema. The first is *histamine*, released by mast cells or basophils. *Histamine* is the main suspect mediator in allergic reactions, since angioedema can be seen in anaphylaxis [1] or as a concurrent symptom of the mast-cell-driven diseases like chronic spontaneous urticaria [2]. For angioedema with unknown aetiology (idiopathic angioedema), histamine receptor antagonists are clinically applied on a trial-and-error basis, sometimes with higher than recommended doses [2, 3]. Approximately one in six patients with idiopathic angioedema remains unresponsive to antihistamines [4, 5]. In such cases, the involvement of other mediators should be considered.

**Fig. 1:**
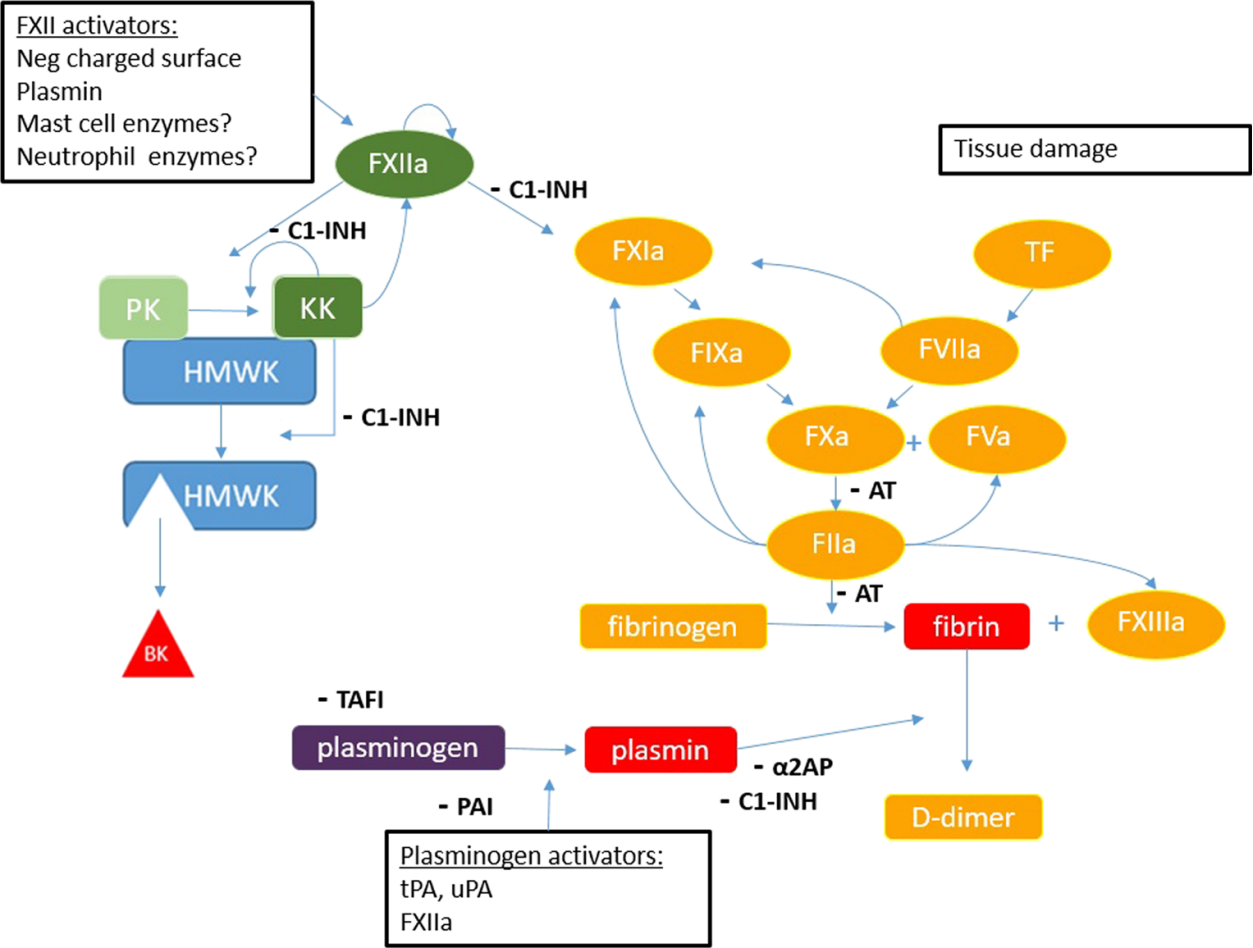
Overview of coagulation, contact activation and fibrinolysis. The coagulation cascade is initiated by either tissue factor (TF) or FXIIa. Positive feedback by *thrombin* (FIIa) accelerates coagulation. The end-product of coagulation is *fibrin*, cross-linked by factor XIII. *Fibrin* is degraded by *plasmin*. During this process, D-dimer, an important clinical biomarker for thrombosis, is generated. Fibrinolysis is started when tPA bound to *fibrin*, or uPA expressed on the endothelium, converts *plasminogen* into *plasmin*. Contact activation starts with the activation of FXII that will eventually lead to *bradykinin* release and vascular leakage. *C1-INH* is the most important inhibitor of contact activation and a weak inhibitor of *plasmin. Anti-thrombin* (AT) inhibits coagulation and *α2-antiplasmin* inhibits *plasmin*; complexes between enzymes and these inhibitors can be measured in plasma as biomarkers for contact activation, coagulation and fibrinolysis. PAI-1 inhibits *plasminogen* activation via inhibition of tPA and uPA. TAFI modulates plasminogen to prevent activation. Abbreviations: *TF tissue factor, FXIIa activate factor XII, PK plasma prekallikrein, KK kallikrein, HMWK high molecular weight kininogen, BK bradykinin, C1-INH C1 inhibitor, AT anti-thrombin, α2AP α2-anti-plasmin, TAFI thrombin activatable fibrinolysis inhibitor, PAI plasminogen activator inhibitor, tPA tissue plasminogen activator, uPA urokinase plasminogen activator*

The second suspect mediator of angioedema is *bradykinin*. This vasoactive peptide was first identified as a mediator for angioedema in patients with hereditary angioedema (HAE) [6–8]. Bradykinin is the end-product of the contact activation system. This enzymatic cascade circulates in the plasma and consists of factor XII (FXII), plasma *prekallikrein* (PPK) and *high molecular weight kininogen* (HK). This system is linked to the intrinsic coagulation system via factor XI (FXI). Classically, the contact activation system is considered to be a redundant part of the blood coagulation system. In vitro, FXII auto-activates when it binds to negatively charged surfaces such as glass or kaolin, hence the name ‘contact system’. Active FXII (FXIIa) activates PPK (Fig. 1). When activated, *plasma kallikrein* (PK) liberates *bradykinin* from HK by cleavage. At present time, it is unknown how *bradykinin* is produced in the human body. Several studies suggested potential natural activators of FXII [9–13], but thus far none of these have been definitively established to induce activation of the contact system during angioedema in vivo.

Here, we will discuss the role of *bradykinin* in angioedema, the link to the coagulation system and how *bradykinin* may be produced in vivo.

## Bradykinin-Mediated Angioedema

The available genetic evidence of HAE-related mutations clearly points towards a central role of the plasma contact system in this disease. Most HAE patients have SERPING1 gene mutations (encoding for C1-inhibitor (*C1-INH*) production) [14, 15] while a small minority have mutations in the F12 gene, with normal *C1-INH* activity [16–20].

Hereditary angioedema is hallmarked by recurrent attacks of angioedema. Attacks can be life-threatening when swelling compromises the airways, and extremely painful when located in the intestine [21, 22]. Therapy targeting the contact system has been successful in HAE, strongly supporting the concept that angioedema is mediated via *bradykinin* production [23–25]. Evidence for *bradykinin* involvement in angioedema is not limited to HAE. First, a comparable phenotype can be observed in patients that have acquired C1-INH deficiency due to underlying auto-immune or lymphoproliferative disease [26, 27]. Second, anti-hypertensive drugs that inhibit *bradykinin* breakdown, such as *angiotensin-converting enzyme* (ACE), *dipeptidyl peptidase IV* (DPPIV) or *neprilysin* (NEP), can induce angioedema. During clinical trials of NEP inhibitors [28], up to 2.17 % of patients and 0.2–0.65 % of patients prescribed ACE inhibitors developed angioedema [29, 30].

Evidently, contact activation is closely linked to the coagulation system [31–33]. Activation of coagulation and fibrinolysis during HAE attacks has been repeatedly reported [34–47]. Yet, HAE patients present with swellings but not with thrombotic tendency [37]. Combined genetic and clinical findings suggest that a subset of coagulation factors are actively involved in angioedema attacks.

## Blood Coagulation

Coagulation factors are readily available throughout the blood circulation to initiate *fibrin* formation and reinforce platelet plugs at sites of injury. These interactions are essential to ensure a properly functioning hemostatic system (Fig. 1). This system consists of a set of precursor proteins (zymogens) that circulate in the blood and has to be activated to become biologically active. The key initiator of the coagulation system, *tissue factor* (TF), is normally not present in the circulation. Cells that surround the vessel wall express TF so that only when the endothelial layer is compromised will locally active coagulation take place [48, 49]. After binding to TF, activated *factor VII* (FVII) of the extrinsic pathway activates *factor X* (FX), and to a lesser extent *factor IX* (FIX) [50]. Activated FX next triggers the formation of a small amount of active *factor II (thrombin). Thrombin* accelerates coagulation via positive feedback mechanisms. *Factor VIII* (FVIII), activated by *thrombin*, forms a complex together with FIX that strongly increases additional FXa and thrombin generation. *Thrombin* activates *factor V (* FV) that, in a similar manner, contributes to thrombin formation in complex with FXa. Furthermore, FXI of the intrinsic pathway is also activated by *thrombin* [51], and additional FIX is being amplified by *thrombin* activation. Ultimately, *thrombin* converts *fibrinogen* into fibrin. *Fibrin* strands are reinforced through cross-linking by activated *factor XIII* (FXIII) (also activated by thrombin). The *fibrin* lattice, together with platelet aggregates and trapped red blood cells, forms a thrombus to seal the damaged area and prevent further haemorrhage. The initiator of this cascade of events, TF, is of vital importance since TF deficiency is lethal and incompatible with normal embryonic development or may cause early death in the perinatal period [52].

### Factor XII as a Coagulation Factor

When blood comes into contact with negatively charged surfaces, FXII activates spontaneously. FXIIa subsequently activates FXI, thereby providing an alternative trigger for coagulation that is independent of TF—the intrinsic pathway. FXII does not take part in the positive feedback mechanism of thrombin. As a result of the discovery of this in vitro mechanism, FXII is generally regarded as a coagulation factor.

Mysteriously, FXII deficiency is a seemingly asymptomatic condition in both mice and human (Hageman’s disease), as it is not associated with a bleeding tendency, unlike deficiencies of other coagulation factors [53]. The same holds for the other components of the contact system cascade; their deficiency does not result in bleeding [54]. This makes the relevance of FXII and the contact system for physiological hemostasis debatable. This logic raises a pertinent question: should FXII be regarded as a coagulation factor, as far as angioedema is concerned? It has been demonstrated that the bradykinin-producing contact system machinery can be fully activated in plasma without evidence of coagulation [10, 11, 55]. This might be explained by the physical properties of FXII(a): in a sequence of cleavage events, FXIIa is progressively fragmented. The fragment that remains after multiple cleavages (βFXIIa or FXIIf) has lost its potential for coagulation while its bradykinin-forming potential remains intact.

### Factor XII as a Bradykinin-Generating Factor

Although the relevance of FXII for coagulation and hemostasis seems to be limited to in vitro experiments, this is certainly not the case for bradykinin production. Low levels of *bradykinin* are continuously formed and present in the bloodstream. Murine models of FXII deficiency indicate that the basal production of bradykinin in plasma is approximately 50 % dependent on FXII [56]. This suggests the presence of a second pathway generating *bradykinin* in vivo. Several alternative, FXII-independent routes for initiation of bradykinin generation have been identified. The endothelial cell-derived factors *heat shock protein 90 (HSP90)* and *prolylcarboxypeptidase* may facilitate FXII-independent PPK activation on the endothelial cell surface [57]. Moreover, *bradykinin* can be directly released from HK by other enzymes than PK; this was recently demonstrated for *Mannose Binding Serine Protease 1* [58]. The relevance of these FXII-independent pathways of *bradykinin* generation needs to be established. It is imaginable that during endothelial cell activation or damage, small amounts of PPK are activated by these mechanisms, which boost up FXII activation as a result.

The clinical importance of FXII for bradykinin generation was already demonstrated in HAE patients. Genome-wide screening of a subset of HAE patients with normal *C1-INH* level and function (formerly called type III HAE) resulted in the discovery of disease-related mutations in the F12 gene (named FXII-HAE). Since then, several mutations in HAE patients have been described, (mostly) located in the *proline*-rich region of FXII (according to mature amino acid sequence: Thr309Arg, Thr309Lys, Ala324Pro, 72-bp deletion at c971_1018þ24 and an 18-bp duplication c894_911, and c1681-1G/A in intron 13) [16–20, 59]. Additionally, isolated cases of normal C1-INH with FXII mutation have been successfully treated with the bradykinin-receptor antagonist—icatibant.

### Plasminogen Activation and Fibrinolysis

After a thrombus has fulfilled its hemostatic function, it has to be cleared. This natural process is mediated mainly by the breakdown of *fibrin*: fibrinolysis (Fig. 1). *Plasmin*, the central enzyme of the fibrinolytic system, cleaves the *fibrin* lattice into smaller *fibrin* degradation products (FDPs) including D-dimers, which are regarded as a valuable biomarker for thrombosis [60]. *Plasmin* is generated from its zymogenic precursor plasminogen by either *tissue plasminogen activator* (tPA) or *urokinase plasminogen activator* (uPA) [61]. *Fibrin* provides a platform for its own degradation by binding and potentiating both tPA and plasminogen. The fibrinolytic system is amenable to inhibition at several stages: *plasmin* is directly inhibited by *α2-antiplasmin*, while specific inhibitors of *plasminogen activators* (PAI-1 and PAI-2) control tPA and uPA [61, 62]. Activation of *plasminogen* on the *fibrin* lattice is indirectly prevented by *thrombin*-activatable fibrinolysis inhibitor, via removal of C-terminal lysines that are needed for the binding of tPA and *plasminogen* to *fibrin*. Intriguingly, *plasminogen* activation on the endothelium can take place in the absence of *fibrin* when the uPA receptor is expressed [63]. This, amongst others, occurs during tissue injury and hypoxia and makes it attractive to speculate that *plasmin* may have additional functions beyond fibrinolysis.

## A Functional Link Between Plasminogen Activation and Contact System Activation

As formerly discussed, the most important function of FXII appears to be activation of PPK. Besides that, FXII is also capable of activation of *plasminogen* [33, 64, 65]. Compared to tPA and uPA, FXIIa is a relatively weak *plasminogen* activator. However, population studies have proposed that FXIIa may protect against cardiovascular disease via *plasminogen* activation [66, 67]. This may be attributable to additional interactions between the contact system and the fibrinolytic system: FXIIa can enzymatically inactivate PAI-1 [68], whereas PK stimulates uPA activation on the endothelium [65].

### Evidence for Plasmin as Natural FXII Activator

There is also evidence that the fibrinolytic system triggers the activation of the contact system. It has been demonstrated that *plasmin* can induce FXII activation in vitro [69]. In line with this finding, patients with myocardial infarction treated with therapeutic *plasminogen*-activating agents, such as streptokinase or recombinant tPA (r-tPA), showed increased plasma levels of *cleaved HK* (a surrogate marker for bradykinin release) [69] and elevated plasma levels of FXIIa [70]. Other clinical observations also support the importance of *plasmin* as a natural FXII activator. Neurologists repeatedly reported angioedema, which is presumably mediated via *bradykinin*, as a side effect of *plasminogen* activators given to patients with ischaemic stroke [71–82]. Up to 8 % of stroke patients receiving r-tPA develop angioedema, often located in the oral cavity and lingual region and contralateral to the infarction site [83]. Similar observations were made with other thrombolytic agents [84–86]. A study with 42 post-r-tPA angioedema cases reported that five patients required emergency intubation or cricothyroidotomy due to laryngeal swelling, with fatal outcome in two cases. Notably, concurrent use of ACE inhibitors is also reported in patients who developed angioedema during r-tPA treatment [78, 79, 83]. Evidence for *plasmin*-dependent bradykinin generation as a cause of angioedema during treatment with fibrinolytic agents is accumulating. However, the majority of these adverse reactions are still treated as a *histamine*-driven hypersensitivity reaction [86]. Future studies should determine if targeting the contact system is beneficial for treatment of angioedema as a side effect of fibrinolytic therapy. Putting these data together makes a strong case for *plasmin*-dependent activation of FXII in vivo.

### The Involvement of Plasmin in HAE Attacks

We recently investigated three subtypes of FXII-HAE patients with F12 gene mutations [87] (Unexpectedly, these patients’ plasma did not enhance FXII-dependent coagulation after contact with a negatively charged compound in vitro (i.e. kaolin). This is in good correspondence with the normal clotting times that were reported in FXII-HAE patients [18]. These patients’ plasma also did not become unusually active upon cleavage by plasma *kallikrein*. Detailed biochemical studies showed that these mutations introduce new cleavage sites in the FXII molecule. The mutated sites are collectively sensitive to cleavage by *plasmin*, resulting in enhanced susceptibility for activation. Analyses of plasma from two FXII-HAE patients who carry the F12 mutation T309K showed that the *plasmin*-forming potential correlated with disease activity. These findings underscore the clinical relevance of *plasmin* as a FXII activator.

Empiric body of evidence for the importance of *plasmin* in the pathology of HAE has been presented in the last four decades. Anti-fibrinolytic therapy, mainly tranexamic acid, has been used as prophylactic therapy for HAE attacks since the 1970s [4, 88, 89]. Tranexamic acid is a *lysine* derivate (analogue) that binds to *lysine*-binding sites of *plasminogen* and thereby prevents its binding to *fibrin* and subsequent activation by tPA or uPA [90]. Moreover, HAE patients with normal *C1-INH* levels (i.e. FXII-HAE) have decreased levels of PAI-2 during remission compared to patients with HAE due to *C1-INH* deficiency [36]. This suggests that their angioedema episodes might have originated from inadequate inhibition of *plasminogen* activation and *bradykinin* formation downstream activated FXII. These findings together point out that *plasmin* formation and contact system initiation might be linked.

## Biomarkers of Contact Activation, Fibrinolytic Activity and Coagulation in Angioedema

### Detection of Contact System Activation

As *bradykinin* was already proven to be the pivotal mediator of angioedema, biomarkers for its production or predecessors are highly sought after, as they may help in diagnosis, monitoring disease activity and response to treatment. *Bradykinin* is only present in the circulation for a few seconds after it is released from HK, due to rapid degradation by *kininases* [91] which complicates its detection. Activation products of contact system activation in plasma are valuable biomarkers as they may reflect recent *bradykinin* production. However, due to pre-analytical in vitro activation, accurate measurements have proved to be challenging and relatively labour-intensive (see Farkas et al., Chapter 7 in this issue).

Circulating *cleaved HK* seems to be among the most suitable biomarkers for contact activation since *cleaved HK* levels have been shown to correlate with attack frequency [92]. Currently, *cleaved HK* can be detected by immunoblotting [92]. This assay often shows profound cleavage of HK in citrated plasma from patients with *C1-INH* deficiency. A diagnostic tool that could specifically measure *cleaved HK*, only after it has released *bradykinin*, would be a compelling instrument for HAE diagnosis and follow-up, but is currently lacking.

### Biomarkers of Coagulation and Plasminogen Activation

There is a large base of evidence that contact activation during angioedema attacks is accompanied by changes in the fibrinolytic and coagulation systems that could serve as disease biomarkers [34–47, 93–95]. The understanding that the contact system and fibrinolytic system are functionally linked sheds a new light on the repeatedly reported occurrence of increased levels of complexes of plasmin and its main inhibitor *α2-antiplasmin* (PAP) and decreased levels of *PAI-1* measured during HAE attacks [34–36, 39, 40, 43, 44]. Fibrinolytic biomarkers may also be helpful in identifying *bradykinin*-mediated angioedema. The presence of these biomarkers in any patient that presents with angioedema in the absence of apparent evidence for thromboembolic event should raise the suspicion that the contact system is involved. Interestingly, parameters of coagulation are repeatedly reported to be increased in HAE. D-dimer levels are elevated during remission periods and markedly increase during attacks [34, 37, 39, 40, 93]. Also, *thrombin-anti-thrombin* complexes and *prothrombin fragments F1+F2* increase during attacks [34, 39, 40, 42, 43, 93].

### The Paradox of Fibrinolysis in HAE

**Fig. 2:**
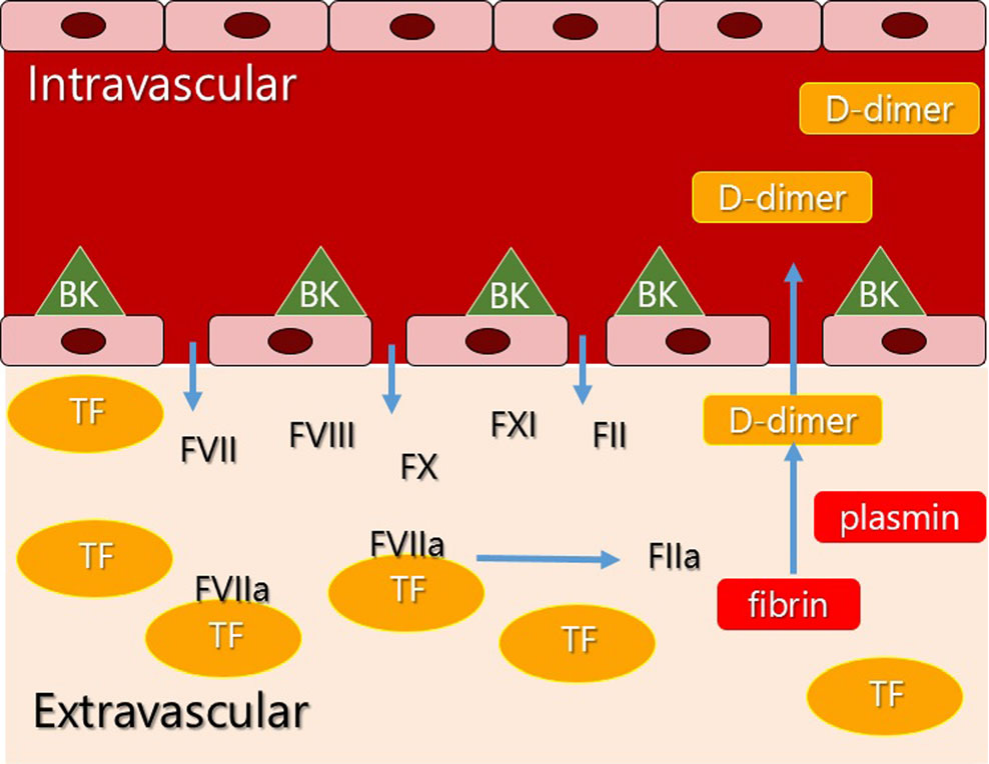
Proposed model: increased plasma coagulation parameters secondary to increased vascular permeability and extravascular coagulation. *Bradykinin* binds to its receptors on endothelial cells. Increased vascular permeability allows extravasation of coagulation factors. Tissue factor expressed in the extravascular space can initiate coagulation. In the absence of any injury, the forming *fibrin* lattice is continuously degraded by *plasmin*. Subsequently, *D-dimer* formed in the extra vascular space dissipates into the blood stream. Abbreviations: *TF tissue factor, FII factor II, BK bradykinin*

Strikingly, HAE is not associated with increased risk of thrombotic disease. It is therefore hard to imagine that the coagulation parameters measured reflect intravascular fibrin formation. Since HAE is associated with extreme vascular leakage, this may offer an alternative explanation for this phenomenon. We would like to propose that when plasma coagulation factors move into the extravascular space, FVIIa may complex with TF. This can trigger coagulation in the absence of vascular injury or intravascular thrombi. Evidence for extrinsic pathway coagulation was demonstrated by a significant increase of FVIIa during angioedema attacks in 14 patients, by Cugno et al. [42]. It would be attractive to hypothesize that increased coagulation parameters in HAE are secondary to massive vascular leakage (Fig. 2). Even though biomarkers for coagulation, such as D-dimer, will not directly reflect *bradykinin* production, their strong association with HAE may make them helpful as biomarkers for monitoring disease activity.

## Future Perspectives in Angioedema—Natural Contact System Activation and Bradykinin Production

Efforts to identify the initial spark of contact activation focused on the presence of in vivo negatively charged compounds (i.e. glycosaminoglycans, misfolded protein aggregates, etc.). The discovery that *plasmin* can enzymatically activate FXII, in the absence of a surface, provides an alternative mechanism for this activation process. The importance is underscored by observations in HAE and patients receiving r-tPA therapy. Under physiological circumstances, this mechanism may take place at sites of vascular obstruction, or slow perfusion, where the tissues are endangered by hypoxia. Alternatively, minor tissue trauma or infection may provide signals to the vascular endothelium, resulting in local sequestration and activation of the contact system. We postulate that FXII functions as a ‘sensor molecule’ that interacts with the environment to detect conditions where increased vasopermeability is required. Excitingly, it is possible that a variety of enzymes other than *plasmin* can fulfil a similar role and contribute to FXII activation.

## Neutrophil Activation and Contact System Activation are Functionally Linked

Recent work shows that HAE attacks are associated with increasing neutrophil counts and neutrophil elastase levels [96]. Neutrophils are important cells for the innate immunity. They migrate out of the bloodstream within hours after a pathogen is detected. Migration of neutrophils to sites of inflammation is orchestrated by several mechanisms, such as chemotaxis by interleukins and complement factors and interaction of neutrophils with endothelial cell receptors [97]. It can be envisioned that neutrophils interact with the contact system to boost neutrophil extravasation by *bradykinin*-mediated vasodilatation. First of all, neutrophil elastase, released by active neutrophils, inactivates *C1-INH* thereby allowing contact activation to take place [98]. Second, in vitro studies show that the *bradykinin B1 receptor* (BKB1R) regulates neutrophil trafficking [99, 100]. Third, both *kallikrein* and FXIIa can induce neutrophil degranulation [101]. Finally, neutrophil extracellular traps (NETs) have been shown to activate FXII. NETs exist of DNA, and the negative charge of DNA is believed to induce auto-activation of FXII [102]. At the very least, NETs can sequester FXII and present it for activating cleavage. NETs are a binding site for antimicrobial proteins such as histones, neutrophil elastase and cathepsins [99]. To what extent the proteins loaded on NETs contribute to neutrophil-induced FXII activation is not yet elucidated. The negative charge of NETs, in combination with potential FXII-activating enzymes, would make neutrophils a plausible platform for contact activation. Further research to neutrophil and FXII activation may help understand its possible relevance for HAE patients.

## Mast Cell Activation Is Linked to Bradykinin Generation

Angioedema as a symptom of anaphylaxis and chronic spontaneous urticaria is likely to be mediated by mast cell/basophil activation. *Histamine* is the central chemical mediator released by mast cells, inducing hyperpermeability and vasodilatation, and is therefore held responsible for the angioedema seen in allergic responses. Indeed, anti-*histamine* therapy reduces the occurrence of angioedema in urticaria patients [2]. It should be noted that plasma *bradykinin* levels were not found elevated in four patients with an acute attack of anti-*histamine*-sensitive angioedema [103]. Yet, evidence accumulates that angioedema in allergic reactions is also accompanied by *bradykinin* release. *FXIIa-C1-INH* complexes and *kallikrein-C1-INH* complexes were increased up to tenfold, within minutes after experimental insect stings in six allergic patients who developed shock or angioedema [1]. In contrast, patients who only developed urticarial rash in reaction to the venom showed a non-significant increase in *C1-INH* complexes. In line with these results, it was shown that in the patients with angioedema or shock, a third up to half of the total pool of plasma HK was cleaved within minutes [1]. These results were confirmed in another study where HK was analysed in patients with an anaphylactic reaction (mainly induced by food allergens) [104]. In this study, even patients with a mild reaction (i.e. gastro-intestinal complaints) showed 60 % cleavage of their HK pool. Evidence of the clinical relevance of *bradykinin* during anaphylaxis with hypotension was further demonstrated in animal models. Hypotension after IgE-mediated, antigen-induced anaphylaxis was reduced in FXII-deficient mice compared to wild-type mice [104]. The same protection toward IgE-mediated hypotension was reported in BKB2R knockout mice and HK- and PPK-deficient mice [105]. Although direct release of *bradykinin* from HK by tryptase could also contribute to these findings, it would be reasonable to propose that *bradykinin* production during anaphylaxis is for a major part FXII-driven.

Moreover, mast cell degranulation may trigger FXII activation via the release of *heparin*, which activates FXII in vitro and induces FXII-dependent hypotension in mice models [10]. *Heparin*-induced vascular leakage in mice was diminished by BKB2R antagonist (icatibant) and exaggerated in *C1-INH*-deficient mice [10]. *Heparin* is a negatively charged compound, capable of inducing FXII auto-activation. However, like the NETs of neutrophils, heparin binds a large variety of proteins [106]. Contrary to this, a study of 14 HAE patients showed that tryptase levels do not increase during HAE attacks [107]. It might be helpful to keep in mind that in HAE patients that also suffer from allergies, attacks may be aggravated by an allergic trigger. What do these studies mean for HAE? Angioedema attacks in HAE are currently not believed to be mast cell-driven (“allergic”), and anti-*histamine* therapy has no effect on symptoms. So far, there is no solid evidence for an association between allergic responses and angioedema attacks. Additionally, *heparin*-protein interactions are usually studied in an isolated in vitro manner. However, the possibility of protein-protein interactions on *heparin* extracellular matrix may be of importance. In the case of FXII, both the net charge of *heparin* and nearby proteins might synergistically activate FXII. Further research into mast cell and FXII interactions may help to understand the pathological mechanisms behind in vivo FXII activation and angioedema.

The contribution of *bradykinin* to angioedema with normal levels of *C1-INH* (i.e. chronic spontaneous urticaria with angioedema, IH-AAE, InH-AAE, U-HAE, etc.) is still uncertain. It might be speculated that since FXII activation can result from mast cell degranulation, *bradykinin* may also play a supportive role in forms of angioedema that are currently classified as ‘histaminergic’, based on the presence of wheals, pruritus or (partial) response to anti-*histamine* therapy. Current studies were unable so far to demonstrate *bradykinin* or an increased HK cleaving in such patients [103, 104, 108]. Considering the strong evidence of interaction between mast cells and *bradykinin* production, patients with idiopathic angioedema and recurrent swellings who fail to respond to high-dose anti-histamine treatment might benefit from therapy targeting the contact activation.

## Conclusions

- FXII is an important player in bradykinin production during HAE attacks. However, it is currently unknown how FXII activation occurs in vivo.
- Coagulation parameters change during attacks. This is possibly secondary to vascular leakage, rather than a consequence of FXII activation. However, biomarkers of coagulation may reflect disease activity.
- Fibrinolytic parameters change during attacks. These may reflect a role for plasminogen activation in *bradykinin* production (via *plasmin*).
- Multiple lines of evidence demonstrate that contact system activation and bradykinin productioncan be triggered by a variety of cell types, including endothelial cells and mast cells.
- It is possible that *bradykinin* plays a role in other forms of angioedema with normal *C1-INH* activity, including those that are currently classified as histaminergic.

## Abbreviations

ACE: Angiotensin-converting enzyme
C1-INH: C1-inhibitor
FVII: Factor VII
FXI: Factor XI
FXII: Factor XII
HAE: Hereditary angioedema
HK: High molecular weight kininogen
NET: Neutrophil extracellular trap
PAI: Plasminogen activator inhibitor
PPK: Plasma prekallikrein
PK: Plasma kallikrein
r-tPA: Recombinant tissue plasminogen activator
tPA: Tissue plasminogen activator
uPA: Urokinase plasminogen activator
TF: Tissue factor
